# Association of HLA-B*5801 allele and allopurinol-induced stevens johnson syndrome and toxic epidermal necrolysis: a systematic review and meta-analysis

**DOI:** 10.1186/1471-2350-12-118

**Published:** 2011-09-09

**Authors:** Ratchadaporn Somkrua, Elizabeth E Eickman, Surasak Saokaew, Manupat Lohitnavy, Nathorn Chaiyakunapruk

**Affiliations:** 1 Center of Pharmaceutical Outcomes Research (CPOR), Naresuan University, Phitsanulok, Thailand; 2 Department of Pharmacy Practice, University of Nebraska Medical Center, Omaha, Nebraska, USA; 3 School of Pharmacy, University of Phayao, Phayao, Thailand; 4 Department of Pharmacy Practice, Faculty of Pharmaceutical Sciences, Naresuan University, Phitsanulok, Thailand; 5 School of Population Health, University of Queensland, Brisbane, Australia; 6 School of Pharmacy, University of Wisconsin, Madison, USA

**Keywords:** Human leukocyte antigen, severe cutaneous reaction, Stevens-Johnson syndrome, toxic epidermal necrolysis, allopurinol, meta-analysis

## Abstract

**Background:**

Despite some studies suggesting a possible association between human leukocyte antigen, HLA-B*5801 and allopurinol induced Stevens-Johnson Syndrome (SJS) and Toxic Epidermal Necrolysis (TEN), the evidence of association and its magnitude remain inconclusive. This study aims to systematically review and meta-analyze the association between HLA-B*5801 allele and allopurinol-induced SJS/TEN.

**Methods:**

A comprehensive search was performed in databases including MEDLINE, Pre-MEDLINE, Cochrane Library, EMBASE, International Pharmaceutical Abstracts (IPA), CINAHL, PsychInfo, the WHO International, Clinical Trial Registry, and ClinicalTrial.gov from their inceptions to June 2011. Only studies investigating association between HLA-B*5801 with allopurinol-induced SJS/TEN were included. All studies were extracted by two independent authors. The primary analysis was the carrier frequency of HLA-B*5801 comparison between allopurinol-induced SJS/TEN cases and each comparative group. The pooled odds ratios were calculated using a random effect model.

**Results:**

A total of 4 studies with 55 SJS/TEN cases and 678 matched-controls (allopurinol-tolerant control) was identified, while 5 studies with 69 SJS/TEN cases and 3378 population-controls (general population) were found. SJS/TEN cases were found to be significantly associated with HLA-B*5801 allele in both groups of studies with matched-control (OR 96.60, 95%CI 24.49-381.00, p < 0.001) and population-control (OR 79.28, 95%CI 41.51-151.35, p < 0.001). Subgroup analysis for Asian and Non-Asian population yielded similar findings.

**Conclusion:**

We found a strong and significant association between HLA-B*5801 and allopurinol-induced SJS/TEN. Therefore, HLA-B*5801 allele screening may be considered in patients who will be treated with allopurinol.

## Background

Stevens-Johnson syndrome (SJS) and toxic epidermal necrolysis (TEN) are severe manifestations of cutaneous hypersensitivity reactions affecting approximately 0.4 to 6 persons per million populations each year [1-3]. Despite the low incidence, the mortality rate has been estimated at 5% for SJS and 30-50% for TEN [2,4,5]. Clinical presentation of SJS and TEN is characterized by a rapidly progressing, blistering exanthema accompanied by mucosal involvement and systemic symptoms that may present as fever, mild elevation of hepatic enzymes, intestinal and pulmonary manifestations [6].

Medications are thought to be a major cause of SJS/TEN cases (~80%). Chemical exposures, mycoplasma pneumonia, viral infections, and immunizations have also been implicated [2]. Iatrogenic causes that have been firmly correlated with both SJS and TEN syndromes include: antiepileptic drugs, antibiotics, and uric acid lowering agents [2,5]. A multinational case-control study recently reported that allopurinol, a xanthine oxidase inhibitor commonly used to treat hyperuricemia and gouty arthritis, was the most frequent drug associated with SJS and TEN [7].

The pathogenesis of allopurinol-induced SJS and TEN is consistent with a delayed-type immune-mediated reaction [2]. The pathogenesis, in conjunction with observed familial predispositions to allopurinol-induced SJS/TEN, alludes to potential genetic-based immunological markers [8]. A number of gene-association investigations have been conducted to elucidate the genetic component of allopurinol-induced SJS/TEN. Results from these studies suggest a strong association with the human leukocyte antigen (HLA), HLA-B*5801 [9-15]. However, these previous studies have shown considerable variation among the magnitude of the association between allopurinol-induced SJS/TEN and HLA-B*5801. A major limitation of the individual studies stems from the low incidence of allopurinol-induced SJS/TEN, which generates observational studies with relatively small sample sizes and insufficient power.

Given the recent accumulation of genetic association studies, inconsistent results, and inadequate power, a quantitative synthesis of the evidence is warranted. The objective of this review is to systematically accumulate and quantitatively analyze the genetic association between HLA-B*5801 and allopurinol-induced SJS/TEN, as well as to elucidate any between-study heterogeneity.

## Methods

### Data sources and search strategy

We performed systematic searches on the following databases: MEDLINE, PreMEDLINE, Cochrane library, International Pharmaceutical Abstracts (IPA), Excerpta Medica Database (EMBASE), Cumulative Index to Nursing and Allied Health Literature (CINAHL), World Health Organization (WHO) International Clinical Trial Registry, and ClinicalTrials.gov from its inception until June 2011. The search terms used were *"HLA-B" *OR *"Human leukocyte antigen" *with AND *"allopurinol" *AND *"Stevens Johnson Syndrome" *OR *"Toxic Epidermal Necrolysis" *OR their acronyms. The Medical Subject Headings (MeSH) was employed when searching database with such option available. Language of the published papers was not restricted and only human studies were included. Bibliographies of the included articles were examined to identify additional studies.

### Study selection

Two reviewers independently evaluated titles and abstracts retrieved from the comprehensive searches for inclusion. Disagreements were resolved by group consensus. To be included, studies were required to meeting the following criteria: 1) investigated the association between HLA-B*5801 and allopurinol-induced SJS/TEN; 2) reported data sufficient for calculating carrier frequency of HLA-B*5801 among cases and controls; 3) cases were subjects that were defined according to detached body surface area as SJS (< 10%), SJS/TEN overlap (10-30%) and TEN (> 30%) [16-18]. Animal studies, case reports, review articles, and duplicate studies were excluded.

### Data extraction and quality assessment

All articles were extracted independently by reviewers (RS, EEE), and discrepancies were resolved through discussion. The following data were extracted from each study: study design, eligibility criteria, diagnostic criteria of SJS or TEN, selection of cases and controls, patient demographics, genotyping technique, and main results. We used Newcastle-Ottawa quality assessment scale [19] for assessing the quality of included observational studies in this review.

### Data analysis

The overall odds ratios (ORs) with corresponding 95% confidence intervals (CIs) were calculated to determine the association between the presence of HLA-B*5801 in at least one allele and allopurinol-induced SJS/TEN. In some studies [11,12], allele frequency data were presented. Based on a study of Tanaka et al [20] and database of Major Histocompatibility Complex [8], we converted allele frequency data into the number of patients with HLA-B*5801 present in at least one allele. All analyses were performed using DerSimonian and Laird method [21] under a random-effects model. Sensitivity analyses were also performed to determine the robustness of the findings by ethnicity. The analyses were also performed separately for those using different types of control groups (e.g. controls obtained from the study, controls obtained from the population database). Statistical heterogeneity was assessed via the Q-statistic and *I*-squared tests [22]. A Q-value of 0.10 indicated statistically significant between-study heterogeneity and *I*-squared values < 25% denoted no/minimal heterogeneity across studies. Begg's test and Egger's test were used to evaluate the publication bias [23,24].

## Results

### Study selection

**Figure 1:**
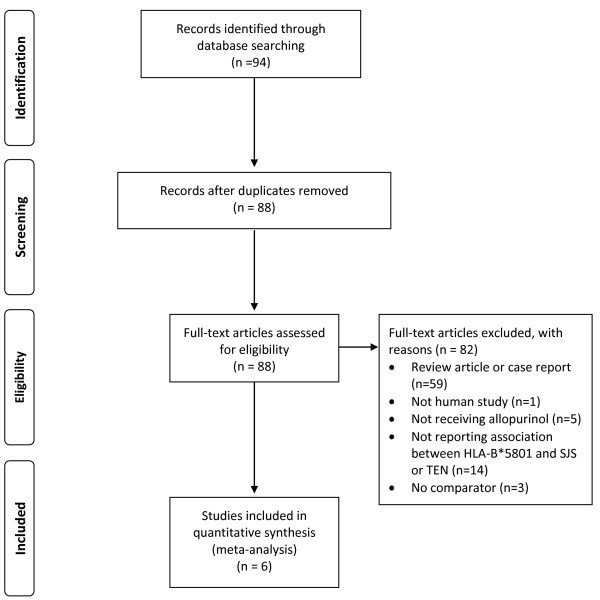
**A flow diagram for study identification, inclusion and exclusion**.

A total of 94 articles were identified. After exclusion of duplication (6 articles), review articles or case reports (59 articles), non-human studies (1 article), studies in which patients did not receive allopurinol (5 articles), studies which did not examine the association between HLA-B*5801 genotype and SJS or TEN outcomes (14 articles) and studies which did not have comparator (3 articles), 6 remaining studies included in the meta-analysis [10-15] (Figure 1). No additional articles were identified via review of the bibliographies of included studies.

### Study Characteristics

Characteristics of included studies are summarized in Table 1 and Table 2. All studies [10-15] included 96 SJS/TEN cases, 678 matched-controls and 3378 population-controls. The reported average age was 57.4 years old (range from 50.0-70.9 years) [10-15] and 51.6 years old (range from 35.9-63.5 years) [10,13-15] for cases and matched-control, respectively. Most patients were men (58.3% for cases and 77.7% for matched-controls). Kaniwa et al [11] and Lonjou et al [12] did not report gender percentage and mean age in the control groups.

**Table 1:** Characteristics of studies included in the meta-analysis

Author	Year	Study Design	**SJS/TEN Cases**^**a**^ (n)	Controls		Data Collection		**Specific Requirement for Case Allopurinol-Exposure**^**b**^	Matching Criteria	NOS
							
				Matched (n)	Population (n)	SJS/TEN Cases	Controls			
Hung SI [10]	2005	Case-control	21	135	93^c^	R	R	Yes	Drug, hospital	7
Kaniwa N [11]	2008	Case-population control^d^	10	-	493^e^	R	-	NR	-	3
Lonjou C [12]	2008	Case-population control^d^	31	-	1822^f^	R (N = 70), P (N = 80)	-	Yes	-	3
Tassaneeyakul W [13]	2009	Case-control^g^	27	54	-	R	R	Yes	Drug, hospital	7
Kang HR [14]	2011	Case-control	5	57	485^h^	R	R	Yes	Drug, hospital	4
Jung JW [15]	2011	Retrospective cohort	2	432	485^h^	R	R	Yes	Drug, hospital	6

Abbreviation: R = retrospective, P = prospective, NOS = Newcastle-Ottawa Scale, NR = not report

^a ^Only patients that received drugs of interest (Allopurinol) were included.

^b ^Including: maximum time to develop adverse drug reaction (ADR) from drug initiation [10,12,13], improvement of symptom upon drug discontinuation [10,13], and exclusion of patients without symptoms upon re-exposure [10].

^c ^Healthy subjects randomly selected from a biobank under nationwide population study, in which 3312 Han Chinese descendants were recruited based on the geographic distribution across Taiwan. There was no self-report of adverse drug events by any of the population control.

^d ^Using population based as control group.

^e ^Data of healthy Japanese reported by Tanaka H, et al. [20].

^f ^Using allele frequencies from European populations available on dbMHC: http://www.ncbi.nlm.nih.gov/projects/mhc/ihwg.cgi

^g ^Author described the study as cross-sectional case-control study.

^h ^Using allele frequency from the general population of Korea.

**Table 2:** Patients' demographic information

Author	Year	% Male		**Mean age**, yr (range)		Ethnicity	Country	Allopurinol Dose, mg/day (range)		Actual Duration of Allopurinol Exposure	
					
		SJS/TEN Cases	Controls	SJS/TEN Cases	Controls			Cases	Control	Cases	Control
Hung SI [10]	2005	52.4	92.6^a^	62.4 (25-91)	56.0 (21-84)^b^	Han Chinese	Taiwan	100 (50-300)	150 (100-400)	26 d (~1-56 d)	22 m (6-107 m)
Kaniwa N [11]	2008	80.0	NA	70.9 (53-83)	NA	Japanese	Japan	NR	NR	NR	NR
Lonjou C [12]	2008	58.1	NA	55.0 (21-83)	NA	Mixed European Population^c^	NR	NR	NR	NR	NR
Tassaneeyakul W [13]	2009	55.6	79.63	65.0 (38-81)	63.5 (46-90)	Thai	Thailand	NR	NR	14 d (3-50 d)	26 m (3-600 m)
Kang HR [14]	2011	60.0	64.9^d^	50.0 (42-80)	51.0 (20-76)^d^	Korean	Korea	200 (100-300)	100 (50-200)	0.7 m (0.2-1.1 m)	29.1 m (6-72 m)
Jung JW [15]	2011	43.8	73.6^d^	41.4 (27-56)	35.9 (18-54)^d^	Korean	Korea	112.5 (74-151)	100 (50-150)	59 d (14-105 d)	887 d (66-1708 d)

Abbreviations: SJS = Stevens-Johnson Syndrome, TEN = Toxic Epidermal Necrolysis; NR = No Report; NA = Not Applicable; d = day(s); m = month(s)

^a ^The percent male of matched-controls (% male in population controls is 55.91).

^b ^Mean age in matched-controls (mean age in population controls is 53.9 (22-91)).

^c ^Including Asian, South American, African, and European.

^d ^The values from matched-controls group.

All SJS/TEN cases in all 6 studies were diagnosed according to the consensus definition [16-18]. All cases required confirmation of diagnosis by dermatologist [10,13], allergy specialist [15], Japan Severe Adverse Reaction (JSCAR) research group [11], or RegiSCAR expert committee [12]. All studies, except Kaniwa et al [11], required specific criteria for allopurinol exposure for the case definition. They included the duration of exposure to allopurinol no longer than 42 days [12], 60 days [15], or 3 months [10,13], and improvement of symptoms after drug discontinuation [10,13]. Hung et al [7] was the only study specifying that patients without symptoms upon re-exposure must be excluded. Despite a requirement of being exposed to allopurinol of cases, three studies [11-13] reported no information on dose of allopurinol and four studies [10,13-15] did not reported the actual duration of allopurinol exposure in both cases and controls.

Three studies [10,13,14] employed classical case control approach in which controls were matched on hospital. In addition, all controls must have used allopurinol at least 6 months without the evidence of any cutaneous reactions. Five studies [10,12-15] also conducted a case control study but used general population as a control group (Table 1). Five studies [10,11,13-15] examined subjects of Asian descent, whereas Lonjou et al [12] examined mixed European population. Hung et al [10] studied Han Chinese population in Taiwan, while Kaniwa et al [11] and Tassaneeyakul et al [13] investigated in Japanese and Thai populations, respectively. In addition, Kang et al [14] and Jung et al [15] studied in Korean population.

All studies determined HLA-B*5801 in cases using PCR-based genotyping and sequence-based typing methods. Since two studies [11,12] used population-controls, there were no descriptions on how genetics of controls were assessed. All studies [11-15] used blood as samples for genotyping, except Hung et al [10] which did not report the source of genetic material. None of the included studies described a blinding procedure for personnel performing genotyping.

### Quality assessment

The methodological quality of all studies was shown as mean Newcastle-Ottawa scale of 5 (range from 3 to 7; maximum possible score, 9) (Table 1). Four studies [10,13-15] using matched controls had scores range from 4-7, while the other two studies [11,12] utilizing population controls received lower quality score of 3.

### Quantitative synthesis

Four studies with 55 SJS/TEN cases and 678 matched-controls were included in the comparisons of carrier frequency to examine the gene association between cases and allopurinol-tolerant controls [10,13-15]. For these 4 studies, the number of HLA-B*5801 carriers was 54/55 and 74/678 among cases and controls, respectively. Five studies [10-12,14,15] with 69 SJS/TEN cases and 3378 population-controls were included in a separate analysis of carrier frequency to test the association of the HLA-B*5801 genotype and allopurinol induced-SJS/TEN compared to the general population. Cumulative carrier frequency for these three studies was 50/69 for cases and 171/3378 for population-controls, respectively.

**Figure 2:**
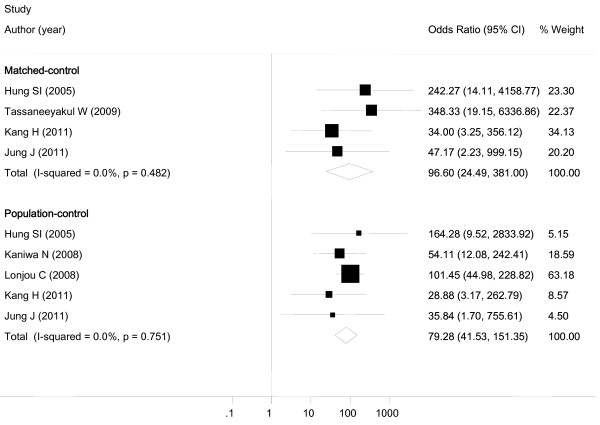
**Forest plot**. A forest plot demonstrating the association between HLA-B*5801 and allopurinol-induced SJS/TEN in matched- and population-control of included studies.

Table 3 summarizes the results of all comparisons. All studies demonstrated a statistically significant association between HLA-B*5801 and allopurinol-induced SJS/TEN. In the primary analyses, SJS/TEN cases were significantly more likely to carry HLA-B*5801 allele compared with both matched-control (OR 96.60, 95%CI 24.49-381.00, p < 0.001) and population-control (OR 79.28, 95%CI 41.51-151.35, p < 0.001) (Figure 2). There was no apparent publication bias as revealed by Begg's and Egger's test (Egger's test for bias, p = 0.732 and Begg's test for bias p = 0.602). No statistically significant heterogeneity was found based on *I*-squared (*I^2 ^*= 0.0%) and Q-statistics (p = 0.482 for matched-control and p = 0.751 for population-control).

**Table 3:** Number of patients who had HLA-B*5801 allele positive and summary odds ratios

Author	Year	HLA-B*5801 Positive/Total		Odds Ratio (OR)	95% Confidence Interval
				
		SJS/TEN Cases (n)	Controls (n)		
**Matched-control**					
Hung SI [10]	2005	21/21	20/135	242.27	14.11-4158.76
Tassaneeyakul W [13]	2009	27/27	7/54	348.33	19.15-6336.86
Kang HR [14]	2011	4/5	6/57	34.00	3.25-356.12
Jung JW [15]	2011	2/2	41/432	47.17	2.23-999.15
***Pooled OR***				***96.60***	***24.49-381.00***
**Population-control**					
Hung SI [10]	2005	21/21	19/93	164.28	9.52-2833.92
Kaniwa N [11]	2008	4/10	6/493	54.11	12.08-242.41
Lonjou C [12]	2008	19/31	28/1822	101.45	44.98-228.82
Kang HR [14]	2011	4/5	59/485	28.88	3.17-262.79
Jung JW [15]	2011	2/2	59/485	35.84	1.70-755.61
***Pooled OR***				***79.28***	***41.53-151.35***

Abbreviations: SJS = Stevens-Johnson Syndrome, TEN = Toxic Epidermal Necrolysis; OR = Odds Ratio

### Sensitivity analyses

Subgroup analysis for Asian and non-Asian population cohorts revealed similar findings. Analyses demonstrated a statistically significant association between allopurinol-induced SJS/TEN with the summary odds ratio of 74.18 (95%CI 26.95-204.14) and 101.45 (95% CI 44.98-228.82) for Asian and non-Asian populations, respectively.

## Discussion

Our findings indicate that HLA-B*5801 allele is significantly associated with increased risk of developing SJS/TEN in patients using allopurinol. This severe adverse event associated with allopurinol could be prevented if such genetic information is known a *priori*. Clinicians and policy makers may use our findings as a foundation to support the implementation of genetic testing prior to initiation of allopurinol.

These findings reveal that the risk of developing SJS/TEN among those allopurinol users with HLA-B*5801 is significantly increased by 80-97 times compared to those without the gene. The sensitivity analyses suggested that the summary odds ratios remained significant regardless of populations. These findings are suggestive of the potential of genotyping in a wide range of population.

Several strengths of our research work deserve more discussion. First, our study is the first one including all kinds of studies determining association of HLA-B*5801 and SJS/TEN development. Second, all SJS/TEN cases were in accordance with the consensus definition [16-18]. These stringent inclusion criteria lowered the risk of misclassification, resulting in increased reliability of our research findings. Third, our meta-analysis adopted the Newcastle-Ottawa scale [19] as a tool to evaluate quality of all case control studies. The Newcastle-Ottawa approach has been reported in several articles to have a good validity for assessing the observational study [25-28]. The average quality score of 5 represented a good quality of overall evidence.

Meta-analysis is not only pooling studies' findings, but this analysis can also determine heterogeneity occurred among the selected studies. The results from our sensitivity analysis demonstrated no significant heterogeneity among populations despite differences in their allele frequency between Asian and non-Asian. Thus, it is justified to perform such analysis despite similarity in the trend of results from the chosen reports. Our findings revealed that despite some differences in several characteristics (e.g. race, sources and selection of control), the association is still consistent and suitable for pooling using meta-analytic technique [29].

Despite the absence of statistical significance of publication bias tests, a possible existence of publication bias cannot be excluded. Because of a relatively small number of total sample size and limited number of studies, the power of publication bias tests might not be sufficient. Even though the meta-analysis shows a consistent significant association of HLA-B*5801 and SJS/TEN in all studies, the overall estimates should be interpreted with caution.

One interesting debate ongoing regarding association between HLA-B*5801 on the risk of developing SJS/TEN is its nature. It has been proposed by Hung et al [10] that HLA-B*5801 was necessary but might not be the only factor related to the risk of SJS/TEN development. Lonjou et al [12] stated that the gene was an important element, but it was neither necessary nor sufficient. All cases in three studies [10,13,15] had HLA-B*5801 allele, whereas the prevalence ranged from 40-80% among cases in the other 3 studies [11,12,14]. The lack of HLA-B*5801 in some cases in the latter 3 studies made it clear that such gene was not the sole factor required. We believe that SJS/TEN development is multifactorial. Other factors including other genes and environment might have a role in the development. What we can conclude was consistent with the summary of Hung et al [10], which was that HLA-B*5801 had a significant role in SJS/TEN occurrence. More research is necessary to further elucidate on how this gene and other factors are involved in allopurinol-induced SJS/TEN pathogenesis.

The increased risk of developing SJS/TEN in those subjects with HLA-B*5801 can be explained by the involvement of cytotoxic T-cells and amplification following cytolytic cytokine. The pathogenesis of drug hypersensitivity is postulated to have direct involvement with human leukocyte antigen (HLA) genes [9,30]. HLA genes located on the major histocompatibility complex (MHC) region of the human chromosome 6p21.3, play a central role in the immune reaction by presenting an antigen to the T cell receptor (TCR) [30].

Despite several postulated mechanisms for the association of HLA-B*5801 and allopurinol-induced SJS/TEN, thus far, there is no definitive proven mechanism. Nonetheless, an immunologic mechanism might play a role in SJS/TEN development [31-33]. Several protein molecules play an important role in this complex process. For instance, MHC proteins, T-cell receptors and some cellular drug metabolizing enzymes (i.e. cytochrome P450 and Phase II metabolizing enzymes) can contribute to the hapten formation and ensuing immunological responses [34]. Although, when the hapten is completely developed and presented at the surface of a nascent T-cell and B-cell clone, they need to proliferate and fully exert their cellular actions. Thus, the fate of these cells can determine the final outcome [35]. These cells may divide and expand resulting in the final immune responses, or get into apoptosis and die out. To elucidate this clonal selection/expansion event among T-cells and B-cells in the SJS/TEN development, a stochastic behavior of the cells is possible [36]. Therefore, a mathematic model describing clonal selection/expansion processes may be useful [37-39]. Nevertheless, this cellular event of the clonal selection/expansion may contribute to the low incidence of SJS/TEN observed in the general population.

Apart from the fate of the nascent antigen specific T- and B-cells, downstream events after binding between the HLA-B*5801 and its receptor may influence the T-cell stimulation. As HLA-B*5801 is presented at the surface, it requires T-cell receptor to couple with the antigen. Subsequently, the immunological system is stimulated [10,30]. The lack of SJS/TEN development might be explained by a malfunction of the T-cell receptor, which could be due to T-cell receptor polymorphism [40,41]. Another putative cause might be an existence of a gene exerting inhibitory effect, resulting in lower risk of SJS/TEN development. In a study of Alfirevic and colleagues [42] investigating the association between genes and SJS/TEN among carbamezepine (CBZ) users, despite small sample size, among those whose possess HLA-B*0702, a significant protective effect for the development of severe reaction was reported. The mechanism of this protective effect is not fully understood.

The association between HLA-B*5801 allele and allopurinol-induced SJS/TEN is consistent across different populations, both Asian and non-Asian [10-15], whereas, an association between HLA-B*1502 and CBZ-induced SJS/TEN demonstrated less consistency [12,43,44]. HLA-B*1502 allele, whose an association with CBZ-induced SJS/TEN is significant in most Asian populations, but not in Japanese and European population. These discrepancies might be explained by the different genetic background. Since this gene is also present in many populations (i.e. African, Caucasian, and Asian), therefore, the association of HLA-B*5801 allele with allopurinol-induced SJS/TEN can be found in various ethnic groups. On the other hand, HLA-B*1502 allele is only present in limited populations (i.e. Asian population) [45].

Interestingly, HLA-B*5801 has a more pronounced effect on allopurinol-induced SJS/TEN compared to those found in the case of HLA-B*1502 and CBZ-induced SJS/TEN. In the latter case, the incidence may be associated with other contributing factors (i.e. other genes) to trigger the adverse drug reaction, whereas those factors may play less role in initiating SJS/TEN in case of HLA-B*580. A study in Japan [46] reported that CBZ-induced SJS/TEN was associated with HLA-B*1511, a member of HLA-B75 type that also includes HLA-B*1502, HLA-B*1508, HLA-B*1515, HLA-B*1521, HLA-B*1530, and HLA-B*1531. These suggested that not only HLA-B*1502 but also other HLA-B75 members are risk factors for CBZ-induced SJS/TEN. By comparison, the strong association between HLA-B*5801 and allopurinol-induced SJS/TEN has been validated in different populations and may be a universal phenomenon since it has been identified in all Chinese, Japanese, Thai, Korean and European patients [10-15].

Notably, a main caveat in this study is the potential misinterpretation of our research findings. This meta-analysis revealed the significant association of HLA-B*5801 allele and the increased risk of allopurinol-induced SJS/TEN. This does not mean that having HLA-B*5801 test done will result in absolutely no risk of allopurinol-induced SJS/TEN. Monitoring of signs and symptoms in these patients are still needed.

Our study is also only limited to the investigation of the association between HLA-B*5801 and allopurinol-induced SJS/TEN. In fact, there has been a number of studies reporting a potential association of DRESS (Drug Rash with Eosinophillia and Systemic Symptoms) and HLA-B*5801 [10,47]. The interpretation of our findings should be limited to SJS/TEN cases and not be generalizable to other severe cutaneous adverse reactions (SCARs).

The implications of our research findings are more likely significant among population with high prevalence of HLA-B*5801. Based on the strong association of the presence of HLA-B*5801 alleles and SJS/TEN, it is presumed that the attributable risk of SJS/TEN due to the existence of this gene is larger among those with the gene. Allele frequency was reported as high as 6-8% among Southeast Asian population and < 1% among Western European population [8,45]. From our results, a genotypic testing of the HLA-B allele may have a benefit to the patients before receiving the drug; particularly in high risk population (e.g. Asian). Knowing the HLA-B allele status of allopurinol users may guide clinicians in determining the optimal choice in order to lower the likelihood of allopurinol-induced SJS/TEN. Presumably these events are avoidable; it might be prudent to consider whether such genetic test should be adopted into routine practice in high-risk population. In order to convince the policy makers to support such genetic testing, a formal cost-effectiveness analysis is warranted.

## Conclusions

We found a strong association between HLA-B*5801 allele and allopurinol-induced SJS/TEN in both Asian and non-Asian population. Knowing the HLA-B allele status may be beneficial to some groups of patients who are about to start receiving allopurinol as such information may help clinicians in determining the optimal drug therapy.

## Competing interests

The authors declare that they have no competing interests.

## Authors' contributions

RS, EEE and SS searched and retrieved data, performed the statistical analysis, interpreted results, and drafted the manuscript. ML interpreted results and drafted the manuscript. NC participated in the concept and design of the study, interpreted results, and drafted the manuscript. All authors read, revised and approved the final manuscript.

## Pre-publication history

The pre-publication history for this paper can be accessed here:

http://www.biomedcentral.com/1471-2350/12/118/prepub
